# Standardization of body weight distribution using a Force-Sensitive Resistor (FSR) matrix and foot analytics

**DOI:** 10.1371/journal.pone.0358764

**Published:** 2026-10-01

**Authors:** Tassadaq Hussain, Soltan Alharbi, Ali Tahir, Mutaz Elradi S. Saeed, Usman Masud

**Affiliations:** 1 Centre for AI and Big Data, Namal University, Mianwali, Pakistan; 2 Department of Electrical Engineering, Namal University, Mianwali, Pakistan; 3 Pakistan Supercomputing Center, and PakASIC, Islamabad, Pakistan; 4 College of Engineering Department of Electrical and Electronic Engineering, University of Jeddah, Jeddah, Saudi Arabia; 5 Department of Computer Science, College of Engineering and Computer Science, Jazan University, Jazan, Saudi Arabia; 6 Department of Computer Science, Nile Valley University, Atbara, Sudan; 7 Faculty of Electrical Engineering and Computer Science, PIMSAT, Karachi, Pakistan; Brunel University London, UNITED KINGDOM OF GREAT BRITAIN AND NORTHERN IRELAND

## Abstract

Balanced body weight distribution across both feet is essential for maintaining musculoskeletal health and minimizing postural and ambulatory issues. Conventionally foot weight distribution is measured using specialized clinical equipment which are expensive and limits routine screening. Therefore, in this work, we present a standardized foot weight distribution model having a custom 32×32 force-sensing resistor (FSR) matrix based embedded system. A total of 4,000 volunteers participated in the Physical Fitness Assessment (PFA). Of these, 2,200 who passed the musculoskeletal fitness criteria were selected for foot pressure data collection. From these data, we derived a standard weight distribution pattern across foot regions: heel (45–55%), midfoot (10–15%), metatarsals (17–27%), and toes (8–13%). These standardized weight distribution patterns are used to identify deviations associated with musculoskeletal conditions that support early personalized rehabilitation and training strategies. The proposed model is implemented on a real-time embedded system and cloud platform. The embedded system uses a K230 RISC-V processor and performs real-time data acquisition, on-board data processing, and visualization of foot weight distribution. The application running on cloud platform performs statistical analysis and applies a classical support vector machine (SVM) for classifying abnormal pressure patterns. A Convolution Neural Network (CNN) based model is developed that learns fit and un-fit foot patterns from the 32×32 pressure maps. The model results show improved detection of dysfunctional weight distribution. The CNN approach shows that AI model can perform advanced predictive analytics in gait and posture assessment. The analysis is limited to static standing posture and does not include dynamic gait assessment. The normative ranges are derived primarily from subjects up to approximately 80–90 kg, as no musculoskeletally fit participants were identified in the heavier weight groups (90–120 kg); therefore, generalization to heavier individuals requires caution and further data collection.

## 1 Introduction

The study of human body weight distribution presents how body weight is transferred to the heel, midfoot, metatarsal heads, and toes. It gives useful information about a person’s biomechanics and musculoskeletal health. Balanced foot weight distribution decreases abnormal joint loading and improves movement efficiency, and gives better performance in daily and professional activities. It also helps in analyzing foot and lower-limb problems, guiding posture correction, and recommending rehabilitation exercises [1–3]. On the other hand, an imbalance in foot weight distribution can have negative effects on human activities [4]. For example, in athletics, it may increase the risk of injuries and create compensatory movement patterns that limit performance. In older adults, uneven weight distribution is linked with postural issues, higher fall risk, and joint degeneration. Long-term imbalance can cause knee, hip, and back pain. Therefore, balanced body weight on the feet gives better physical performance and stamina [5].

Different methods and technologies, such as force plates, pressure mats, and wearable insole systems, are used to measure foot weight distribution [6–8]. The approaches use real-time data for both static and dynamic body states. Imaging techniques along with gait analysis are also used to understand the relationship between foot loading and overall human movement patterns [9–13]. However, there is considerable variation in hardware systems, measurement protocols, and analysis methods across different methods and techniques. This lacks consistency and makes it difficult to use the technologies broadly in clinical and field settings [14]. In addition, the existing systems are expensive, not easily available, and require trained users and controlled laboratory environments. Wearable solutions are more practical but often use fewer sensing elements, which leads to lower spatial resolution and limited calibration. Existing studies use small sample sizes, focus on specific groups, and do not provide detailed age- and weight-based reference values [15]. As a result, there is still no widely accepted standard reference for plantar load or foot weight distribution in healthy adults across different body types and conditions.

Therefore, there is a need for a simple and reliable baseline that describes how healthy people distribute their body weight across the feet while standing. This baseline should be created using a large sample of musculoskeletally healthy individuals, measured with a well-defined protocol and a calibrated sensing system. A standard model of foot weight distribution is also required to detect meaningful deviations in different settings, including general health screening, sports performance assessment, and follow-up during rehabilitation or long-term disease management [16–19].

In this work, we address this gap by developing and applying a low-cost, high-resolution measurement system for foot weight distribution. Work includes collecting a large dataset from adults who meet strict musculoskeletal fitness criteria. A custom 32×32 force-sensing resistor (FSR) matrix is used to capture detailed plantar pressure maps and an embedded system is developed to convert sensing values into foot weight distribution patterns. Based on data from 2,200 fit participants out of 4,000 screened volunteers, we derive standard ranges for the percentage of body weight carried by the heel, midfoot, metatarsals, and toes. Which includes a standard weight distribution pattern across foot regions: heel (45–55%), midfoot (10–15%), metatarsals (17–27%), and toes (8–13%). The proposed system is designed to be portable and affordable, making it suitable for use outside specialized gait laboratories. By providing both a standardized reference model and a reproducible embedded system, the work targets more consistent assessment of balance and posture, earlier detection of foot-related abnormalities, and more targeted rehabilitation and training strategies.

## 2 Related work

Previous research on foot load distribution covers several areas, including general weight distribution and biomechanics, measurement technologies for plantar pressure, and applications in gait and posture analysis. In this section, we briefly review these works and highlight the gaps that motivate our study.

Zhang et al. [18] analyzed plantar pressure and weight distribution in healthy adults. The work reports a static body weight pattern in which the heel bears large portion, followed by the metatarsal heads and then the toes. The work showed that the proposed organized weight distribution pattern is linked to the anatomical structure of the foot and supports stable standing. Cerezci-Duygu et al. [4] studied the effects of foot weight distribution in female adolescent athletes using a plantar pressure platform. The work reports that a common pattern holds about 60% of the weight on the rearfoot and 40% on the forefoot. The work also found that increasing forefoot loading can improve postural stability. The work shows how small changes in rearfoot-forefoot distribution can improve the body balance. Ahmad et al. [20] investigated weight distribution on the 5th metatarsal of the right and left foot in young adults. The research found a clear difference between feet and reported a moderate positive correlation between the two sides. The results underline that local asymmetry in body weight distribution is important and relevant for personalized podiatric care and orthotic design. The studies on statistic weight distribution confirm that weight distribution is closely related to posture, balance, and musculoskeletal health. However, it mainly focus on specific regions and groups and do not provide a standard reference pattern for healthy adults.

Vigneshwaran et al. [6] developed foot plantar pressure measurement systems and compared different sensor technologies, including capacitive, resistive, and piezoelectric sensors. The work compared strengths and limitations of existing platforms and proposed a wireless plantar pressure system. The proposed wireless system measure high pressure distributions under the foot with good accuracy. The review compares both the maturity of the field and the cost and complexity of many commercial systems. Mohammed et al. [15] designed a system to determine weight distribution in the foot and to assist in diabetes diagnosis. They combined vibrotactile sensation tests with optical sensing to estimate plantar loading. Their work demonstrated that lower-cost optical and sensory methods can be used to detect abnormal loading in patients with reduced sensation. Godfrey et al. [21] used in-shoe capacitive sensors to compare foot pressure distribution during walking in young and elderly adults. This study highlighted age-related changes in plantar loading. It showed that older adults tend to shift the body weight towards the lateral side of the foot, especially in the heel and hallux regions. Arzehgar et al. [22] developed a wireless insole foot pressure data acquisition device. The device uses capacitive sensors placed at key plantar locations. This work presented a portable, low-cost insole systems to monitor plantar loading outside the laboratory. The work recorded plantar pressure during different physical activities and transmitted data wirelessly to a base station. Carbonaro et al. [23] proposed a low-cost stretchable sensorised sock based on a resistive matrix. Their sensorised sock integrated 32 textile-based piezoresistive sensors under the heel and metatarsal areas to capture plantar pressure during walking. They research presented a strong correlation between their sock and reference systems and demonstrated that textile-based sensor matrices can support daily-life gait analysis. Overall, these works show a clear move from heavy, lab-based force plates towards more portable pressure mats, insoles, and textile socks. At the same time, many systems use different sensor types and calibration methods, and not all studies report detailed calibration, drift correction, repeatability, or cross-validation against gold-standard systems. This lack of consistency makes it difficult to compare results across studies and to define common reference values.

Cerezci-Duygu et al. [4] focused on rear and fore foot weight distribution ratio and its relationship with balance and plantar pressure parameters in adolescent athletes. Their research showed that, while standing, about 60% of body weight is on the rearfoot and about 40% is on the forefoot. The research also showed that increasing forefoot loading can improve balance measures. Their findings highlight the role of static foot loading patterns in functional balance training. Montagnani et al. [24] studied plantar pressure distribution while standing on flat and inclined surfaces. They reported that the right foot often bears more pressure and on a sloped surface the pressure shifts towards the forefoot. Their work presented how surface angle affects the plantar pressure and design footwear and ergonomic interventions. Ahmad et al. [20] researched on foot pressure distribution during walking by young and elderly adults. They presented that differences were concentrated in the calcaneus and hallux regions and on the medial side of the foot. The work concluded that lateral weight bearing in the elderly may reduce gait stability. This work links regional plantar pressure changes with age-related balance and stability issues. Wang [25] proposed a deep learning approach using a Long Short-Term Memory (LSTM) model. The AI model estimates full foot pressure distribution during walking from a reduced set of insole sensors. They showed that the LSTM model can reconstruct detailed pressure maps with good accuracy. The work suggest that advanced AI algorithms can compensate for sparse hardware and provide rich plantar pressure information. Parker et al. [26] performed gait analysis using wearable sensors including: a) accelerometers, b) gyroscopes, c) magnetometers, and d) pressure sensors. They described how these sensors can be combined to capture gait dynamics and discussed the use in clinical and sports applications. The work shows that plantar pressure is one important part of a broader wearable-sensor ecosystem for gait assessment. Wang and co-authors [25] proposed a statistical method for footprint analysis using a large-scale and high-density piezoresistive film. They showed that piezoresistive film can provide detailed spatial maps of plantar loading. The work used statistical techniques for analysis and to support objective footprint classification and assessment.

Together, these studies show that plantar pressure patterns are tightly linked to gait and posture, and that data-driven methods, including classical statistics and neural networks, can be used to interpret complex pressure data and detect abnormal patterns. However, most of these works use limited sample sizes, focus on specific populations, or do not report simple, standard percentage ranges for heel, midfoot, metatarsals, and toes that can serve as a general clinical reference.

In summary, previous research has improved understanding of foot weight distribution and has delivered many useful hardware systems and analysis methods [4,6,15,20–25,27–29]. At the same time, the literature remains fragmented due to differences in hardware, calibration, sample size, and reporting [30–35]. There is still no widely accepted standard model that describes how musculoskeletally healthy adults typically distribute their body weight over the heel, midfoot, metatarsals, and toes during quiet standing [26,36–41]. The proposed work addresses this gap by using a cost-effective, high-resolution 32×32 FSR matrix with a large group of adults who meet strict musculoskeletal fitness criteria. The work applies a structured foot weight distribution protocol having detailed calibration, validation, and consistent data processing. Based on this, it derives standardized foot weight distribution patterns of different foot regions. These patterns provide a practical baseline to support clinical assessment, performance evaluation, and future data-driven research.

## 3 Materials and methods

The data flow shown in Fig 1 and Table 1, describe how system collects, processes, and sends foot weight data. First, a 32x32 flexible FSR matrix (RX-M3232L) is placed under both feet. The sensor converts the pressure at each point into an electrical signal. These signals transfers to Data Acquisition System, which uses a 32:1 multiplexer/demultiplexer and an ADC to scan all 1,024 sensors. At this stage, the system performs basic hardware filtering and preprocessing, and converts the raw signals into 32×32 digital pressure values. Next, the data moves to the Memory System, where a 32x32x1 buffer holds the foot pressure values. The Processing System reads the pressure maps from memory, runs the detailed analytics and prepares the results. Finally, the processed data is sent over Ethernet to the Cloud Application, where the analytics dashboard displays heat-maps, numeric summaries, and reports. The section is further subdivided into (a) participants, (b) hardware and sensor system, (c) data architecture and acquisition, (d) feature extraction and classification, and (e) visualization and cloud deployment.

**Table 1:** Structured Data Architecture for Foot Weight Distribution Analysis.

Attribute	Description
Name	String: Subject Identifier
Age	Float: Years.Months
Weight	Float: Kilograms (kg)
Height	Float: Centimeters (cm)
Dysfunctions	String: Physical Issues
Foot Length	Float: Centimeters (cm)
Foot Width	Float: Centimeters (cm)
Pressure Data	Int: 32×32 Matrix

**Fig 1:**
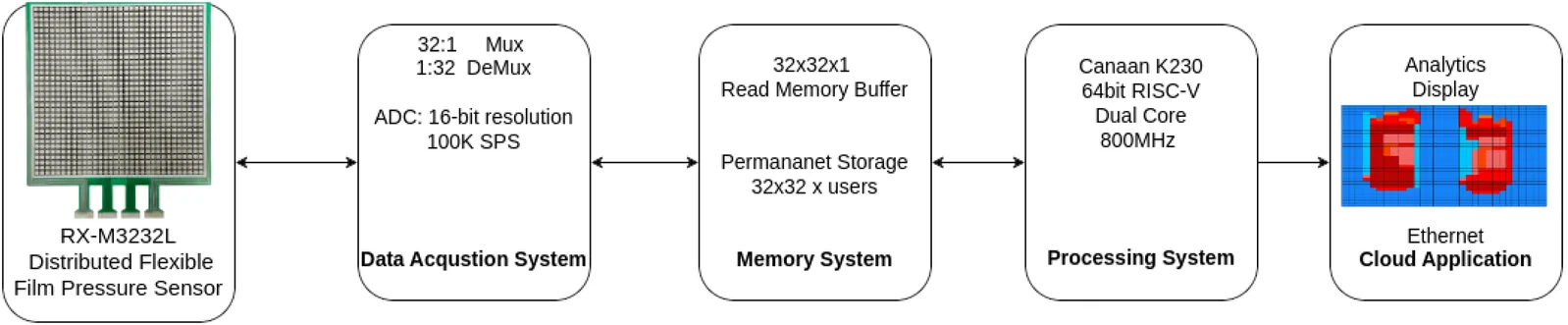
Flow of the foot-weight distribution system, from the 32 × 32 FSR sensor and data acquisition, through local processing and storage, to cloud-based analytics and visualisation.

### 3.1 Participants

This section describes how the participants are recruited and screened for the reference dataset. A total 4,000 adult volunteers (age range approximately 20–60 + years; all above 18 years old) are recruited from the general population. The sample included both male and female subjects with a wide range of body weights and heights, so that the final reference model would reflect typical variability in healthy adults. All participants completed a Physical Fitness Assessment (PFA) before foot pressure measurements. The PFA included:

- Measurement of body mass index (BMI).
- Joint range of motion assessment (e.g., ankle dorsiflexion and knee flexion) using a goniometer.
- Simple strength and endurance tests (body-weight squats and single-leg heel raises).
- Visual posture inspection to check for obvious spinal or lower-limb deformities.

The participants were classified as *musculoskeletally fit* if they met the following quantitative criteria:

- BMI in the normal range (18.5–24.9 kg/m^2^).
- At least $15^{\circ }$ of ankle dorsiflexion and $130^{\circ }$ of knee flexion in both legs.
- Ability to perform at least 20 consecutive body-weight squats and 15 single-leg heel raises without pain or loss of balance.
- Balanced static posture on visual assessment (no marked limb length discrepancy or major spinal deformity).

Out of 4,000 volunteers, 2,200 met all criteria and were classified as musculoskeletally fit. Their data were used to establish the reference foot weight distribution model. The remaining 1,800 participants did not fully meet one or more criteria. These participants were grouped as *musculoskeletally unfit* and their data were used separately for comparison. All participants provided written informed consent before testing. The study protocol was reviewed and approved by the University of Sialkot Research Ethics and Data Access Committee (Approval/Reference No.: **D/EIRB/USKT/22**). The data collection procedures followed the principles of the Declaration of Helsinki. The released data were de-identified (no names, contact information, or national identifiers), and subject IDs were pseudonymous.

### 3.2 Hardware and sensor system

This section explains the sensing mat and embedded hardware used to acquire plantar pressure and process weight distribution respectively shown in Fig 2. The sensing mat uses custom-built 32×32 force-sensing resistor (FSR) matrix sensor array (Rx-M3232L) with a total sensing area of 15×15 inches. The array contains 1,024 sensing elements, each capable of detecting loads from approximately 200 g up to about 100 kg on that cell. This resolution allows detailed mapping of plantar pressure under both feet. The main hardware components are:

**Fig 2:**
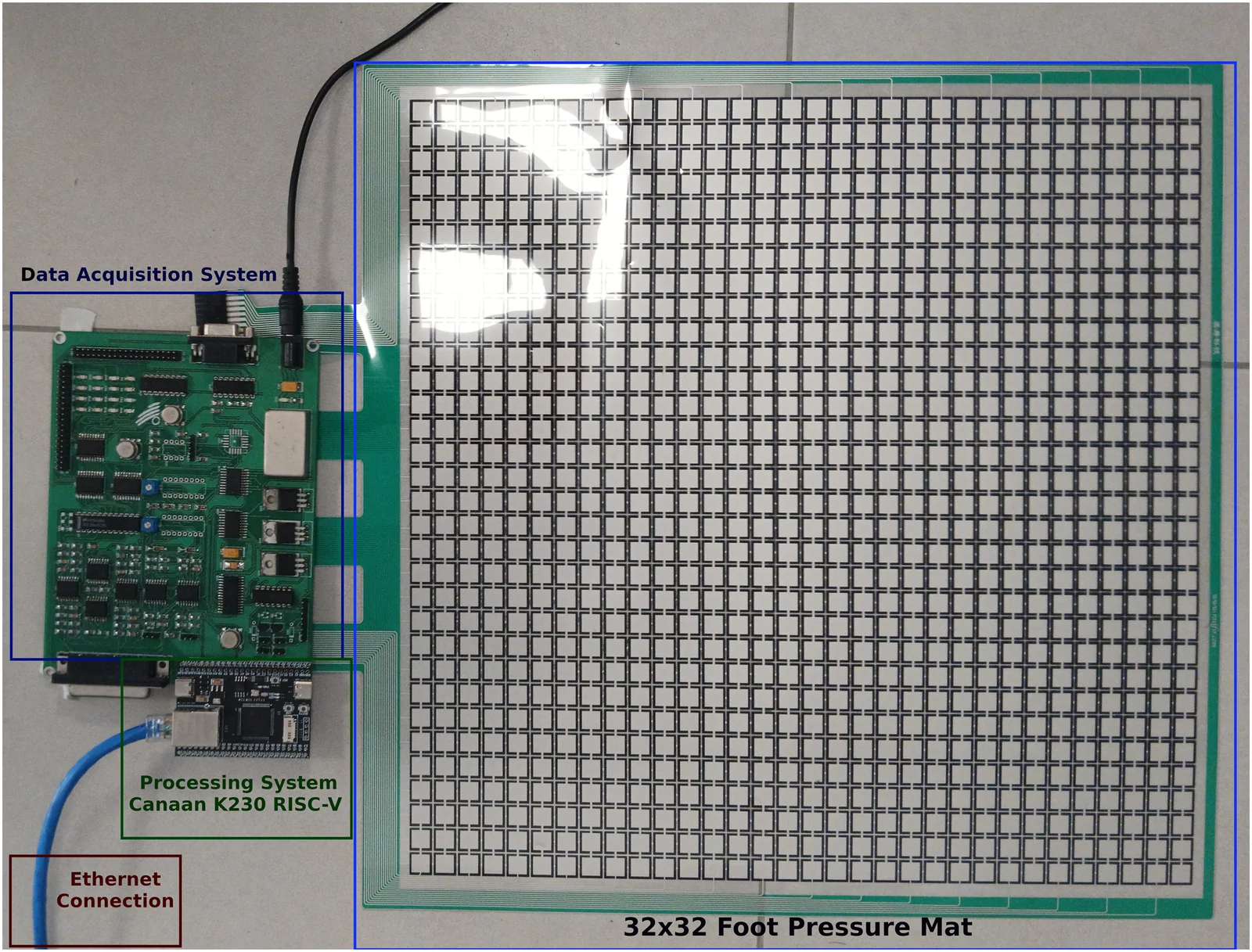
Photograph of the Hardware Prototype for the Foot Weight Distribution Measurement System.

- A 32×32 FSR matrix (Rx-M3232L) to measure plantar pressure.
- An ADG731 multiplexer to sequentially select each sensor cell.
- A Wheatstone bridge and a precision instrumentation amplifier (TI INA333) to convert and amplify the small resistance changes produced by the FSR cells.
- Canaan K230 RISC-V processor based embedded system with a built-in analog-to-digital converter (ADC) for data acquisition and processing.

At run-time the embedded system scans all FSR (32x32) matrix sensors using ADG731 multiplexer and read the foot weight distribution. The Wheatstone bridge and INA333 amplifier convert the change in resistance of each FSR into a voltage signal. The signal is then digitized by the K230 ADC having 12-bit resolution and 100 Hz of sampling frequency. The K230 processor runs application written in C for data logging, analysis, and display. Because FSR sensors have a non-linear response and can drift over time the system performed a detailed calibration and validation procedure:

- **Calibration:** Before data collection 100K ohm resistors were placed against FSR sensors and recorded the raw calibrated ADC values. A calibration curve was derived against each sensor that converts raw readings to force (Newtons) and non-linearity correction.
- **Baseline correction:** At the start baseline values were collected from the sensor unit. To manage the drift the baseline values were subtracted from subsequent measurements.
- **Test-retest reliability:** For a reference the subject stood on the FSR mat ten times in the same position. It was confirmed that the regional load (heel, midfoot, metatarsals, toes) was below 3%.
- **Force-plate validation:** Measurements from 10 participants were recorded on both the FSR mat and a laboratory force plate. This was used to confirm that the total force measured by the FSR mat was within about 5% of the force plate values. The percentage load distribution across regions also showed a strong correlation (*r* > 0.95) between the two systems.

### 3.3 Data architecture and acquisition

This section describes how the raw data are acquired and stored in the foot weight distribution dataset. The data acquisition protocol for body weight distribution uses a static standing posture. Each participant is asked to stand quietly on the sensor mat, without talking, shaking, or unnecessary movements, until the signal is stable. The sensor matrix then records the pressure data for both feet at the same time. The sensor architecture collects raw readings from all 1,024 sensor cells. Each reading is stored as a 16-bit integer, which can represent values from 0 to about 65,000 16-bit un-signed integer formate. These values are later arranged in a 32×32 two-dimensional matrix data-structure, as shown in Eq. (1). This matrix is the basic input structure used for further analysis.


$$\begin{array}{c} 𝐏=[\begin{array}{cccc} P_{1,1} & P_{1,2} & \cdots & P_{1,32}\\ P_{2,1} & P_{2,2} & \cdots & P_{2,32}\\ \vdots & \vdots & \ddots & \vdots \\ P_{32,1} & P_{32,2} & \cdots & P_{32,32} \end{array}] \end{array}$$ (1)

The $P_{i,j}$ (Equation 1) represents the calibrated force at row *i* and column *j*. Each value is stored as a 16-bit integer to keep enough precision for later processing. Based on these input values and the participant information listed above, a complete record is created for each trial. Later, along with the foot weight distribution matrix, a dataset record is created for each participant that includes the following information:

- Demographic information (age, sex).
- Basic anthropometric data (height, body weight, BMI).
- Simple clinical information (history of foot or lower-limb problems, if any).
- Foot measurements (for example, approximate foot length and width).
- Raw plantar pressure data from the 32×32 FSR matrix.

This record forms one entry in the foot weight distribution dataset used for standardization and analysis.

### 3.4 Data processing and region segmentation

This section describes (Fig 3) how raw sensor data is converted into foot weight distribution values and converted according the region of the foot (heel, midfoot, metatarsals, and toes). During acquisition, the K230 processor scanned all sensors at 100 Hz and convert raw ADC readings into forces (Newtons) for each cell. For each trial, the system:

**Fig 3:**
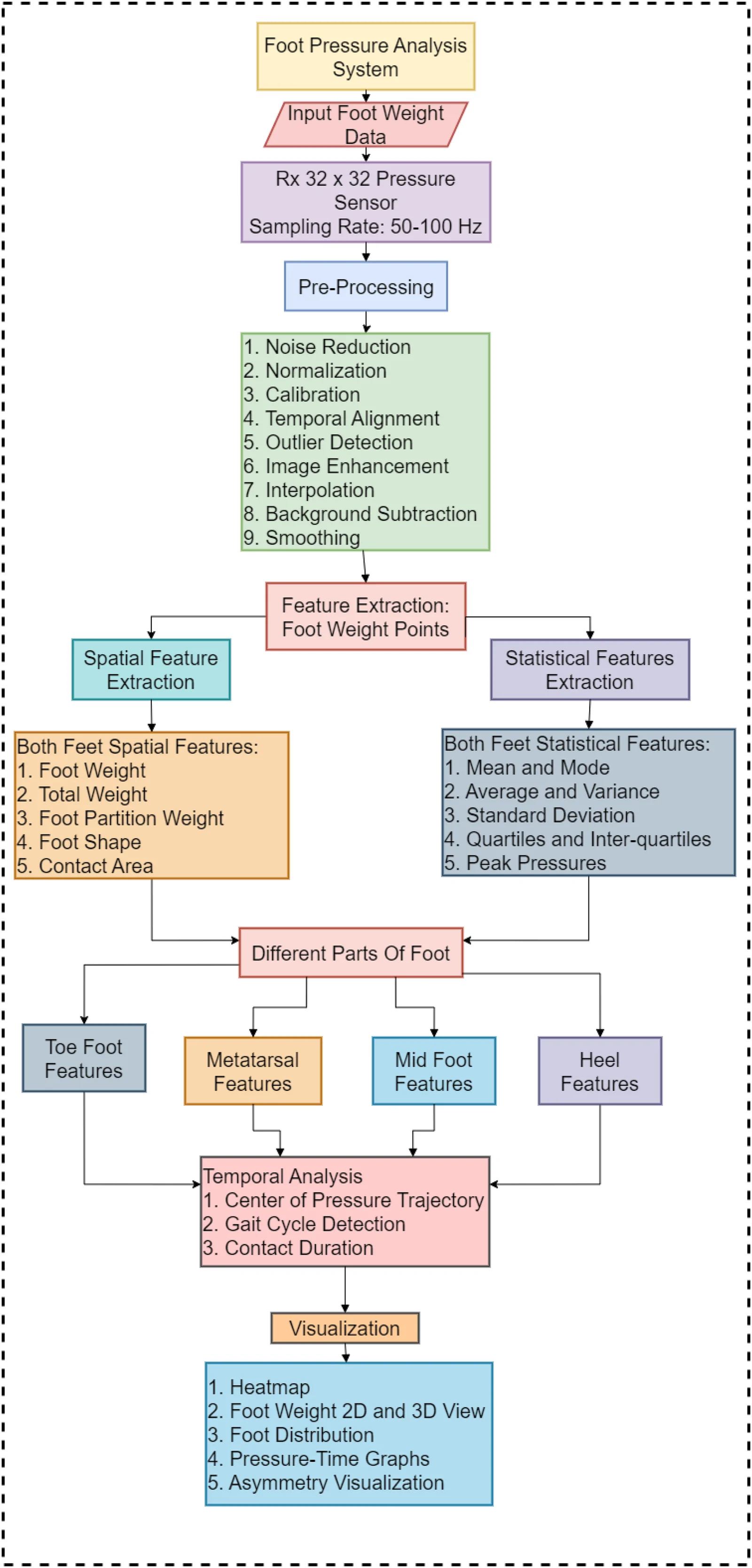
Software processing flow of the Foot Weight Distribution System.

1. Recorded the participant’s body weight using a standard digital scale.
2. Asked the participant to stand upright on the mat, with both feet comfortably placed at shoulder width, looking forward.
3. Collected 5 seconds of steady-state data and computed the average force at each sensor cell.

The data is then processed the averaged force matrix as follows:

- **Foot detection:** A simple threshold is applied on 32x32 2D Vector data-structure (Equation 1) to identify active values which presents the feet.
- **Alignment and footprint extraction:** A bounding box is applied around each foot based on active cells. While using principal component analysis (PCA) footprints are aligned with its long axis.
- **Region segmentation:** Each foot is divided into four anatomical regions along its length: heel, midfoot, metatarsals, and toes. The foot region boundaries are defined using fixed proportions of foot length and visual inspection against anatomical landmarks.
- **Load computation:** For each foot region, the calibrated forces/pressure of all sensor cells are summed up within the region. The regional load is then normalised by the total measured load (sum over both feet) and expressed as a percentage of body weight.

### 3.5 Feature extraction and classification

Features are extracted from the foot weight data and the classification techniques used to separate healthy and unhealthy loading patterns. From each 32×32 foot weight data structure, features are extracted to show (a) how much weight is on each part of the foot. (b) how the weight is shared between the left and right feet, and (c) what the overall footprint looks like in terms of shape and size. While targeting foot weight distribution following features are extracted:

- **Centre of pressure (COP):** The average point of contact for each foot is calculated by taking a weighted average of all sensor positions, where the weights are the local forces. This gives a single point that summarises where most of the load is acting on each foot.
- **Regional weight distribution:** For each of the four regions (heel, mid-foot, metatarsals, toes), the forces of all sensors in that region are summed and then divided by the total measured force. This gives the percentage of body weight on each region and is the main feature used for standardized foot weight distribution.
- **Simple ratios:** Ratios such as rear-foot vs. forefoot load and left vs. right foot load are computed. These ratios give a quick view of how weight is shared between back and front of the foot, and between the two feet, which is useful in clinical screening and sports assessment.
- **Descriptive statistics:** Basic statistical analysis are performed which include the mean, median, minimum, maximum, and standard deviation of the forces. It shows how evenly or unevenly the pressure is spread inside a region and help to identify body weight high-pressure points.
- **Foot and region shape indicators:** The number of active sensors (non-zero cells) in each region is used for contact area estimation. Using this shape-related features such as footprint length, width, and mid-foot contact areas are derived. Later these regions are used to describe body weight distribution. It is also used to describe the foot shape such as: flat, high-arched, or narrow/wide foot shapes in a straightforward numerical form.

To capture overall shape differences in the pressure maps, principal component analysis (PCA) [36] is applied to the flattened 32×32 matrices. PCA reduces the high-dimensional pressure patterns to a small set of components that explain most of the variation in the data. The scores of the first few principal components are then used as compact descriptors of the pressure pattern. This allows the analysis to retain information about footprint shape and detailed loading patterns, while keeping the number of features manageable.

Based on these features, machine learning and deep convolutional neural network-based classification techniques are used. A support vector machine (SVM) classifier [40] is used to distinguish between musculoskeletally fit and unfit subjects. The SVM with a Gaussian kernel is trained using the regional percentages, descriptive statistics, ratios, and PCA scores, with labels taken from the PFA classification (fit vs. unfit). The SVM is a standard method that works well for medium-sized datasets and produces a clear decision boundary between classes.

In addition to the SVM, a simple convolutional neural network (CNN) model is implemented that takes the full 32×32 calibrated pressure map as input. The CNN consists of two convolutional layers with 5×5 kernels, each followed by max-pooling, and two fully connected layers with a final softmax output (fit vs. unfit).

All custom code used for data acquisition, calibration, feature extraction, and classification (including the SVM and CNN implementations), as well as scripts to reproduce the figures, is uploaded as Supporting Information (S1 Data).

### 3.6 Visualization and cloud deployment

This section describes how the foot weight distribution application hanedel data at run-time, using embedded system, cloud computing to perform visualization.

At run-time the subject trial data and its management performed locally on the embedded system and transferred to a secure cloud server. Before upload, all data are anonymised and linked only to a study ID. The cloud platform (shown in Fig 4) takes the pressure maps, weight distribution values, and participant information. It also allows authorized users to run the SVM and CNN models on new data and to track changes over time for follow-up studies.

**Fig 4:**
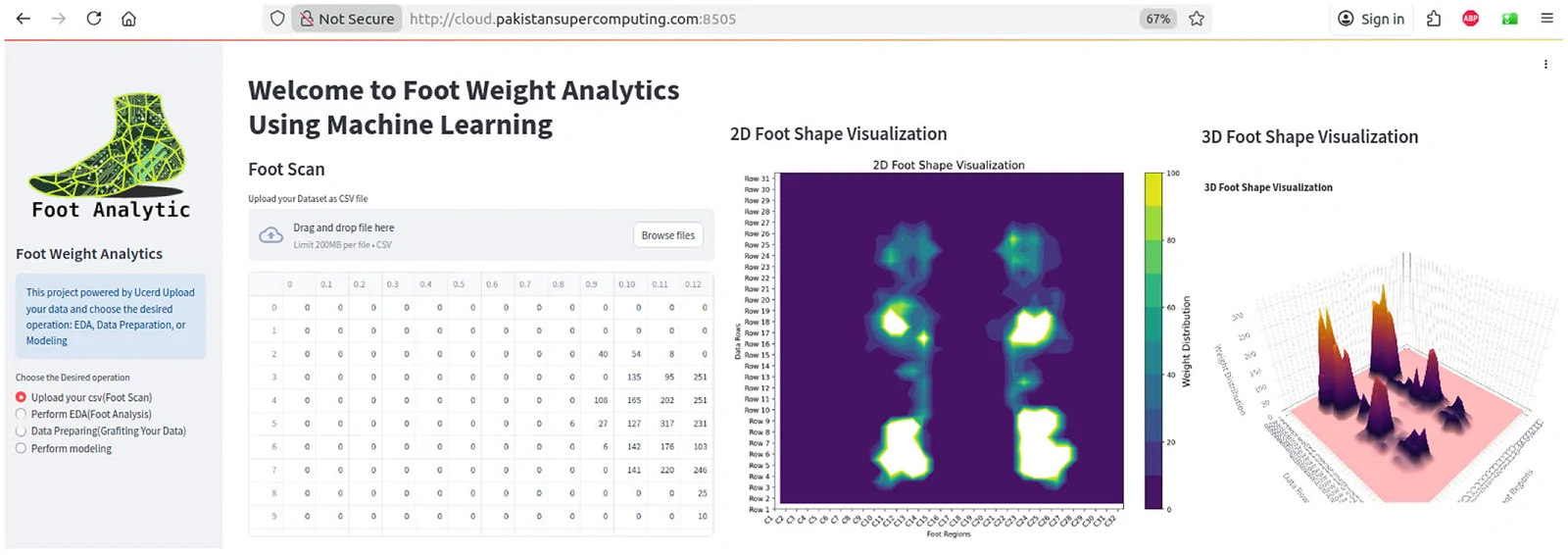
Diagram of the Cloud-Based Analytics Framework for the Foot Weight Distribution System.

At local machine the embedded system also displays summary statistics on a built-in LCD which include the percentage of body weight on the heel, midfoot, metatarsals, and toes for each foot. How ever for advanced analysis cloud application is used. The cloud application gives visual feedback by generating 2D heatmaps and 3D plots (Fig 5) of the 32×32 sensor array, where colour intensity shows the local force level. These heatmaps (Fig 5a) and simple 3D surface plots (Fig 5b) help clinicians and participants to see which parts of the foot bear more weight. This approach allows foot weight distribution system to use in clinics, sports centres, and community settings while still maintaining a central database for research and long-term monitoring.

**Fig 5:**
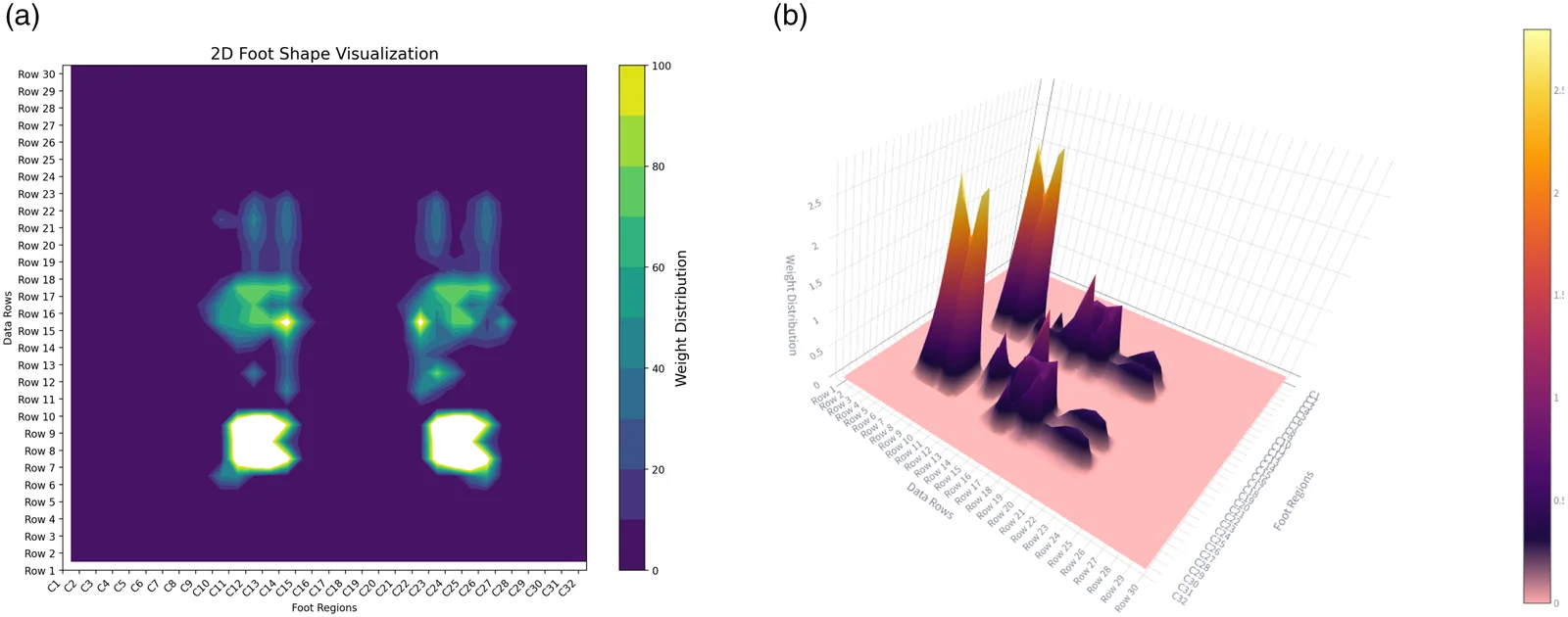
Visualization of foot weight distribution using (a) a 2D heatmap and (b) a 3D surface plot. (a) 2D heatmap of the 32×32 foot weight distribution. (b) 3D surface plot of foot weight distribution for both feet.

## 4 Results

This section discusses the standardized foot weight distribution results obtained while using the working foot weight distribution system shown in Fig 6. It includes: a) Physical Fitness Assessment, b) standardized foot weight distribution in musculoskeletally fit subjects, c) foot weight distribution in musculoskeletally unfit subjects, and d) classification performance and accuracy.

**Fig 6:**
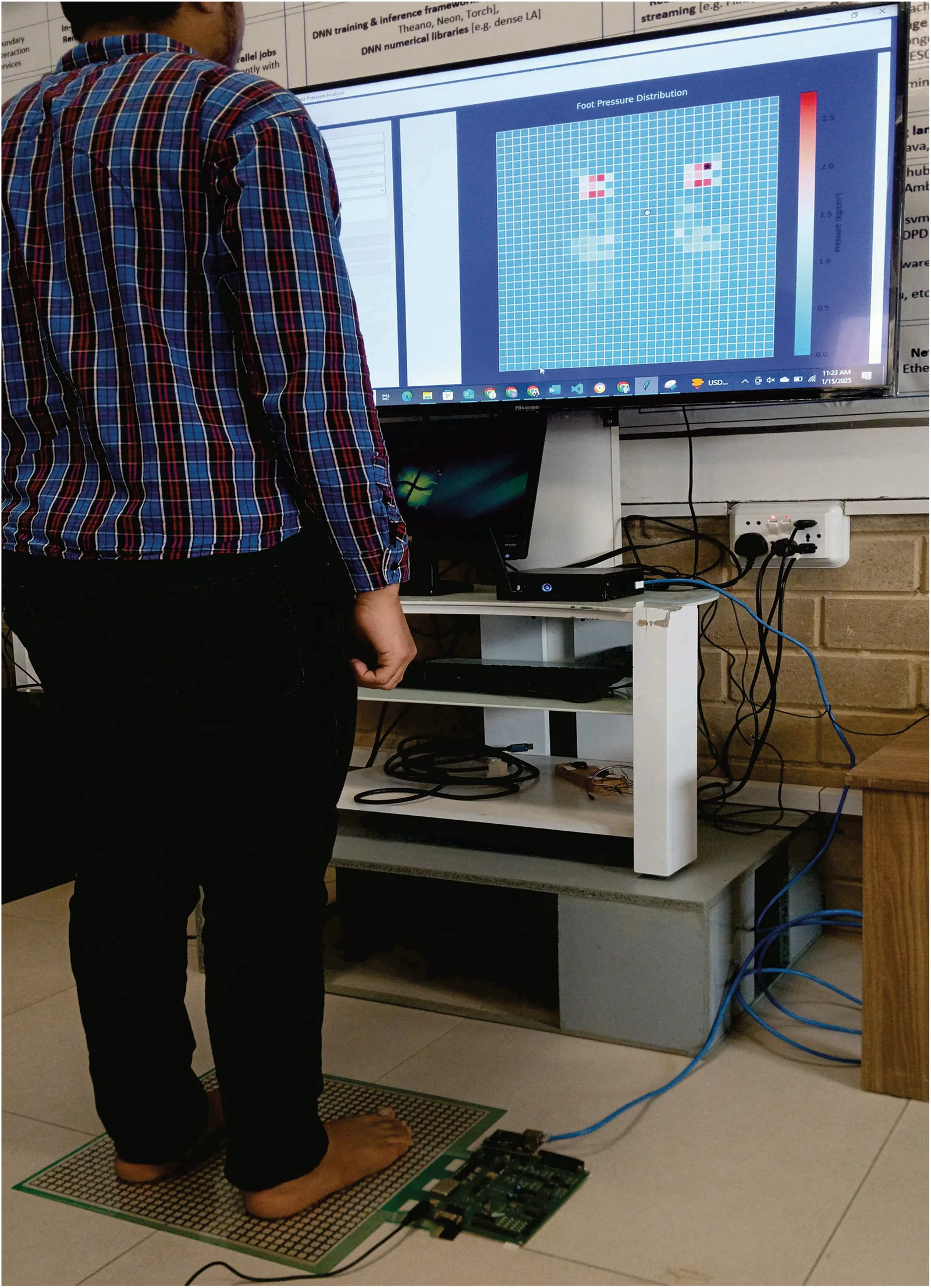
Photograph of the Functional Hardware Prototype of the Foot Weight Distribution System Collecting Data from Subjects.

### 4.1 Physical fitness assessment and subject classification

In this experiment participants physical fitness assessment and classification mechanism is described. A total of 4,000 adults completed the Physical Fitness Assessment. Based on the predefined criteria (BMI, joint range of motion, strength/endurance, and posture), 2,200 individuals met all requirements and were classified as musculoskeletally fit, while 1,800 were classified as unfit.

In general, the fit group showed better mobility, strength, and postural control than the unfit group. On average, fit participants had greater ankle dorsiflexion (often 10–15° more than unfit subjects) and greater hip extension. They could also complete more body-weight squats and single-leg heel raises, indicating higher strength-to-body-weight ratios and better lower-limb endurance. In contrast, many unfit participants had limited ankle flexibility (some could not dorsiflex beyond 5–10°) and showed earlier fatigue during functional tests.

Participants were also organised into 13 weight-height-gender groups (shown in Table 2) to explore trends across body size. In practice, nearly all fit individuals were found in weight ranges up to about 80–90 kg (Groups 1–8). We did not find any musculoskeletally fit subjects in Groups 9–12 (90–120 kg). Many participants in these heavier groups showed increased lumbar lordosis, reduced joint mobility, and lower relative strength, and therefore did not pass the PFA criteria. Group 13 (heaviest and tallest subjects) contained only a small number of volunteers and was not large enough to define a separate stable reference pattern. As a result, the standardized model presented in this paper mainly reflects healthy adults up to approximately the 80–90 kg body-weight range.

**Table 2:** Subject groups categorised by weight, height, and gender.

Group	Gender	Weight Range (kg)	Height Range (ft)
1	Male	50-70	5.0-5.6
2	Female	50-70	5.0-5.6
3	Male	50-70	5.7-6.0
4	Female	50-70	5.7-6.0
5	Male	70-90	5.0-5.6
6	Female	70-90	5.0-5.6
7	Male	70-90	5.7-6.0
8	Female	70-90	5.7-6.0
9	Male	90-120	5.0-5.6
10	Female	90-120	5.0-5.6
11	Male	90-120	5.7-6.0
12	Female	90-120	5.7-6.0
13	Male	90-120	6.0+

### 4.2 Standardized foot weight distribution in fit subjects

In this experiment we described the standardized foot weight distribution model mechanism using the system (Fig 6). The standardized foot weight distribution model was derived from the 2,200 musculoskeletally fit subjects. Foot pressure data were recorded from the custom 32×32 FSR mat during quiet standing. Figs 7 and 8 show representative regional distributions for lighter (50–70 kg) and moderate (70–90 kg) weight groups, while Fig 9 illustrates a typical pattern for one heavier fit subject. Figs 7–9 show the standardized foot-weight distribution of musculoskeletal fit subjects. Each vertical bar represents one subject. The total height of the bar represents the subject’s body weight in kilograms (y-axis). Bars are ordered from lighter to heavier subjects from left to right. The colored segments within each bar show how body weight is distributed across the four foot regions: heel (blue), midfoot (orange), metatarsals (yellow), and toes (green). For example, a 60 kg subject with 30 kg on the heel, 8 kg on the midfoot, 15 kg on the metatarsals, and 7 kg on the toes appears as one 60 kg column split into those four colored parts. In this way, the figures give the actual numeric load (in kg) on each region for every subject included in the plot, and it directly corresponds to the percentage ranges reported for heel, midfoot, metatarsals, and toes. Figs 5a and 5b present 2D and 3D visualizations of the averaged pressure maps. While analyzing all 2,200 fit subjects, the mean regional weight distribution was found as follows:

**Fig 7:**
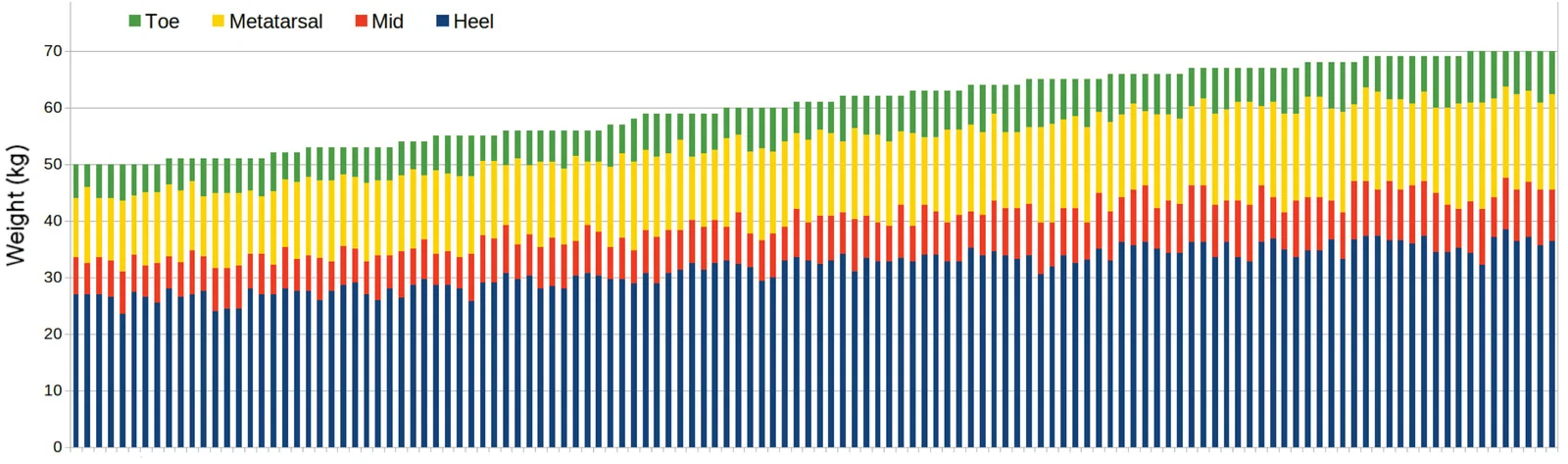
Group 1-4 Stacked bar chart of regional foot weight distribution of Musculoskeletal Fit Subjects. Each bar presents one subject; total bar height is body weight (kg). Colored segments show weight on heel (blue), midfoot (orange), metatarsals (yellow), and toes (green).

**Fig 8:**
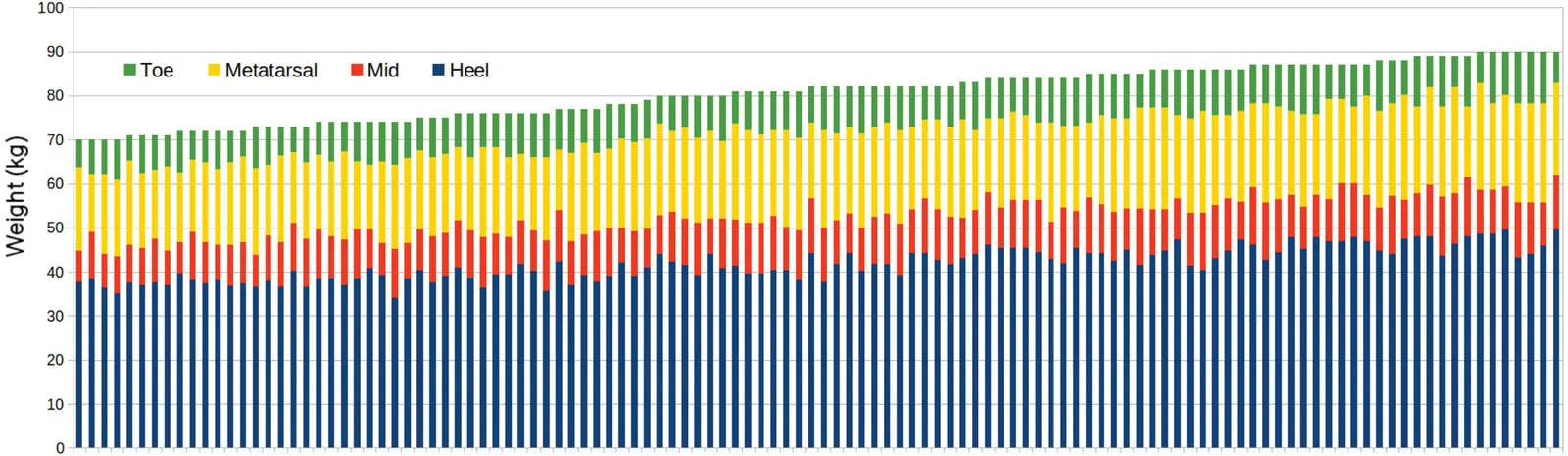
Group 5-8: Stacked bar chart of regional foot weight distribution of Musculoskeletal Fit Subjects.

**Fig 9:**
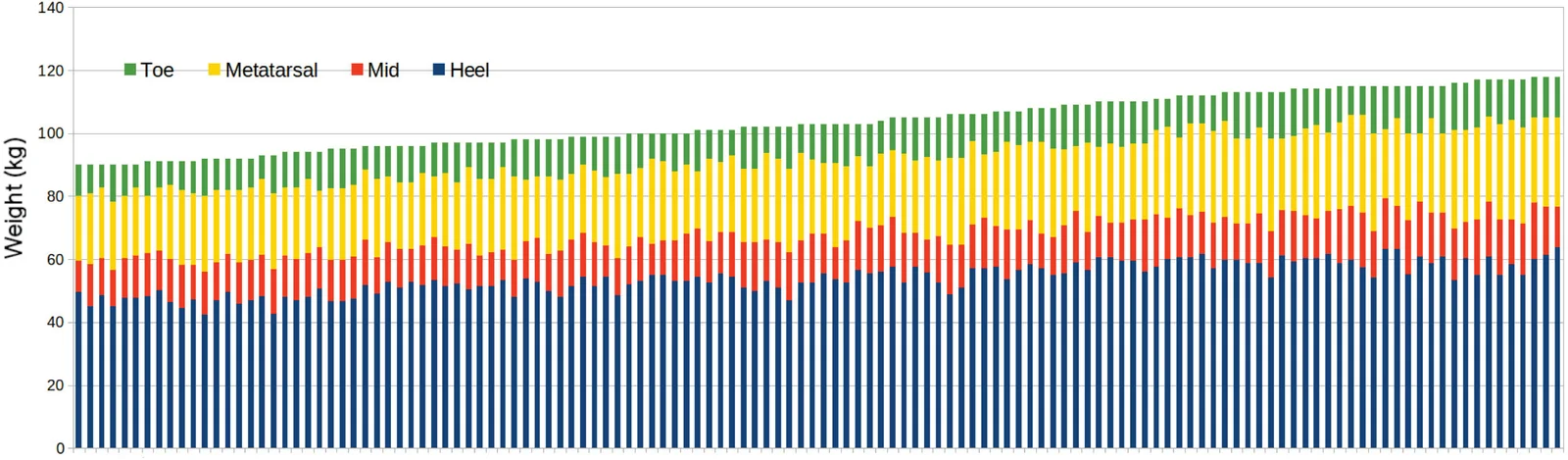
Group 13: Stacked bar chart of regional foot weight distribution of Musculoskeletal Fit Subjects.

- Heel: 51.99% (SD 2.35%)
- Midfoot: 12.97% (SD 1.66%)
- Metatarsals: 24.04% (SD 2.29%)
- Toes: 10.99% (SD 1.63%)

From these results, the typical distribution ranges for healthy adults are summarized as:

- Heel: 45–55% of body weight
- Midfoot: 10–15%
- Metatarsals: 17–27%
- Toes: 8–13%

These values confirm the expected pattern in quiet standing: the heel supports roughly half of the body weight, the midfoot (arch) carries about one-tenth to one-sixth, the metatarsals carry about one-fifth to one-quarter, and the toes carry around one-tenth of the load. The relatively small standard deviations show that, within the fit group, these percentages are quite stable across different body sizes, especially in the midfoot and toe regions. When we compared subgroups by weight, height, and gender, the mean percentages stayed within these ranges, and only small changes in standard deviation were observed. This suggests that the proposed ranges can be used as a practical baseline for musculoskeletally healthy adults, with only minor adjustments needed for extreme body sizes.

### 4.3 Foot weight distribution in musculoskeletally unfit subjects

In this experiment, we discuss how and why a subject is classified as musculoskeletally unfit based on foot weight distribution. Our results show clear deviation of Foot pressure distributions patterns in the 1,800 unfit subjects. In many unfit participants, the heel region (calcaneus) holds high body weight distribution which is between 55% and 65%. This indicates a shift of the centre of pressure towards the rearfoot. This pattern suggests that these individuals rely more on passive heel support and this is likely due to limited ankle dorsiflexion and reduced calf strength, or balance problems. On the other hand the midfoot (arch) region of the unfit subjects is often underloaded. The average values between 2% and 7% of body weight. This reduced loading reflect poor engagement of the medial longitudinal arch which shows a tendency to keep weight behind the midfoot, rather than distributing it along the full length of the foot. The metatarsal region in unfit subjects usually carried between 18% and 22% of body weight. While this range overlaps with healthy values, the pattern was more variable and sometimes unbalanced between the medial and lateral metatarsals, suggesting uneven load transfer across the forefoot.

The toes showed the most marked reduction in loading, with many unfit individuals placing only 3% to 5% of body weight on the toes. This low toe engagement suggests limited use of the toes for balance and push-off, and is consistent with weak plantarflexors, reduced toe joint mobility, or avoidance of forward weight shift.

Taken together, these results indicate that musculoskeletal unfitness is characterized by:

- Inflated rearfoot loading (heel-dominant pattern).
- Underuse of the midfoot arch region.
- Less effective and sometimes uneven forefoot loading.
- Very low use of the toes for support and propulsion.

The standardized ranges derived from fit subjects provide a clear reference to identify and quantify these deviations in clinical and sports settings.

### 4.4 Classification performance

In this experiment, we evaluated how well the extracted features and pressure maps are distinguish between musculoskeletal fit and unfit subjects. Using the regional foot weight percentages and simple statistical features (such as mean and standard deviation in each region), together with PCA-based shape descriptors, a support vector machine (SVM) classifier achieved an accuracy of about 90% for classifying normal versus abnormal loading patterns. This result indicates that the selected regional and statistical features contain valuable information about overall musculoskeletal fitness.

The proposed convolutional neural network (CNN) was trained on the full 32×32 calibrated pressure maps, using 80% of the data for training and 20% for validation. The model achieved an overall accuracy of 94.7% (95% CI: 93.1%–96.2%) on the validation set. The confusion matrix showed 943 true positives, 57 false positives, 912 true negatives, and 48 false negatives for the fit versus unfit classification. Per-class performance was as follows: fit class – precision 94.3%, recall 95.2%, F1-score 94.7%; unfit class – precision 94.1%, recall 93.0%, F1-score 93.5%. These results confirm that the CNN can extract discriminative features directly from raw pressure maps, outperforming the SVM classifier. The higher accuracy of the CNN suggests that spatial patterns beyond simple regional averages contribute to distinguishing fit from unfit loading behaviour.

This approach can be used to identify more body weight distribution dysfunctionalities. The results conclude that the standardized foot weight distribution and statistical features are informative. The information provide support and help in distinguishing healthy from unhealthy loading behavior. From foot weight patterns, SVM provides an interpretable feature-based view. Whereas the CNN shows more advanced data-driven methods.

The main focus of the paper, however, remains the standardisation of regional foot weight distribution ranges in healthy adults. The classification results are presented as supporting evidence that these ranges are meaningful and useful for future analysis and screening.

## 5 Discussion

This section discusses the main findings, possible biomechanical explanations, practical applications of the proposed standard foot weight distribution model, and its limitations and future directions.

The key contribution of this work is the development of a standardized model of foot weight distribution in musculoskeletally fit adults. Using data from 2,200 fit participants, the study identified that, during quiet standing, healthy subjects typically place about 45–55% of body weight on the heel, 10–15% on the midfoot, 17–27% on the metatarsals, and 8–13% on the toes. These values confirm the general understanding that the heel carries roughly half of the body weight in static stance, while the metatarsals and toes support the remaining load in a graded pattern. The ranges are based on a large sample and therefore provide practical reference values for clinical and research use.

The comparison between fit and unfit groups highlights how abnormal loading patterns can develop. Musculoskeletally unfit participants showed a heel-dominant pattern, with 55–65% of body weight on the heels, and much lower loading on the midfoot and toes (often only 3–5% on the toes). This pattern suggests that many unfit individuals avoid shifting the centre of pressure forward. Limited ankle dorsiflexion, stiffness in the calf-Achilles complex, and weak plantarflexor muscles are likely contributors. If the ankle cannot move freely, or if calf strength is insufficient, the body tends to remain “behind” the foot, keeping more weight on the heel and less on the forefoot and toes. Persistent heel-biased loading may increase stress on the heel pad, plantar fascia, knees, and lower back, while reduced forefoot and toe loading may compromise balance and push-off. These findings are consistent with previous reports linking underuse of the forefoot and toes with poorer postural control, and they quantify the difference between fit and unfit patterns in simple percentage terms.

From a clinical perspective, the standardized ranges can serve as a baseline for screening and monitoring. For example, a patient after ankle, knee, or foot surgery can be asked to stand on the sensor mat, and regional percentages can be compared with the reference ranges. Persistent deviations, such as very high heel loading or very low toe loading, may indicate incomplete recovery or a need for specific exercises to restore joint mobility and muscle strength. In chronic conditions such as plantar fasciitis or diabetic foot complications, regular measurement of foot weight distribution may help to detect harmful loading early and to guide orthotic design or physiotherapy programmes.

In sports and performance settings, the model can support identification of athletes with risky loading habits. An athlete who consistently loads far outside the normal ranges (for example, excessive forefoot loading or marked asymmetry between left and right feet) may be at increased risk of overuse injuries in the foot, ankle, or lower limb. The same system can be used to track the impact of training interventions, footwear changes, or taping strategies by observing whether foot weight distribution moves closer to the healthy reference pattern over time.

The hardware and analytical framework used in this study are designed to be practical in real-world environments. The 32×32 FSR matrix provides high spatial resolution with low cost than many commercial force plates. This make it suitable for clinics, gyms, and community centres. Calibration, reliability testing, and comparison with a laboratory force plate indicate that the calibrated FSR matrix can measure total load and weight distribution with accuracy. The support of support vector machine (SVM) and convolutional neural network (CNN) models show that the foot weigth distribution can be used for advanced pattern recognition.

The classification results show that weight distribution patterns are not only descriptive but also useful for intelligent problem identification. Using foot weight regional features and principal component analysis (PCA) scores, the SVM classifier identified fit and unfit subject with an accuracy of about 90%. Whereas the CNN, gives an accuracy of approximately 95%. These findings suggest that deep learning models can capture subtle differences in pressure distribution that are not fully represented by simple regional averages. In future work, similar models could be trained to recognise specific clinical conditions or risk profiles directly from pressure maps. In the present study, however, the primary focus remains on the definition of clear normative ranges.

The current design and methodology have a few limitations, which are documented. First, the analysis was restricted to static standing. Dynamic activities such as walking, running, or jumping produce time-varying pressure patterns that may reveal additional aspects of foot function and injury risk. Extending the protocol to dynamic trials would allow a link between static ranges and gait phases or loading rates. Second, the definition of “fit” was deliberately strict in order to construct a clean reference group. As a result, the presented ranges represent an ideal or best-case pattern. In real-world populations, many individuals will fall slightly outside these ranges without clear pathology. The reference values should therefore be used as a guide and interpreted alongside clinical judgement and other assessments. Third, although calibration and validation were performed carefully, FSR sensors remain sensitive to environmental factors and long-term drift. Regular recalibration and routine quality checks are recommended for long-term deployment. We note that our analysis is restricted to static standing; dynamic gait analysis was not performed. We note that our analysis is restricted to static standing; dynamic gait analysis was not performed. This limitation (lack of gait data) may restrict clinical applicability. Additionally, although our initial recruitment included weight groups up to 120 kg, no musculoskeletally fit participants were identified in the 90–120 kg range (Groups 9–12). This finding limits the generalizability of the normative ranges to heavier individuals and suggests that different fitness criteria or modified protocols may be needed for this population. Future work should consider targeted recruitment of fit individuals in higher weight categories or the development of weight-stratified reference ranges.

## 6 Conclusion

In this work, we proposed a standardized foot weight distribution system using a low-cost 32×32 FSR matrix. The key contribution of the work is the standardization of foot weight distribution patterns for musculoskeletally fit subjects. The data patterns were developed using a low-cost 32×32 FSR matrix and a large cohort of 2,200 fit subjects. The data pattern shows that musculoskeletally fit subjects typically place about half of their body weight on the heel, around one-tenth to one-sixth on the midfoot, about one-fifth to one-quarter on the metatarsal heads, and roughly one-tenth on the toes. These patterns provide reference values to check whether a person’s loading pattern is normal or clearly deviates from it. Second major contribution of the work is distinguishing fit and unfit subjects. Unfit individuals show heel-dominant loading and reduced use of the midfoot and toes. This shows limited forward weight shift, weaker push-off, and poorer postural control. Machine learning SVM and deep learning CNN models are used in the study. The proposed model and system offer a practical basis for objective musculoskeletal assessment.

## Supporting information

S1 Data Data. (ZIP)
